# SNP-SNP Interaction Network in Angiogenesis Genes Associated with Prostate Cancer Aggressiveness

**DOI:** 10.1371/journal.pone.0059688

**Published:** 2013-04-03

**Authors:** Hui-Yi Lin, Ernest K. Amankwah, Tung-Sung Tseng, Xiaotao Qu, Dung-Tsa Chen, Jong Y. Park

**Affiliations:** 1 Department of Biostatistics, H. Lee Moffitt Cancer Center & Research Institute, Tampa, Florida, United States of America; 2 Department of Cancer Epidemiology, H. Lee Moffitt Cancer Center & Research Institute, Tampa, Florida, United States of America; 3 Behavioral and Community Health Sciences, Louisiana State University Health Sciences Center School of Public Health, New Orleans, Louisiana, United States of America; 4 Department of Biomedical Informatics, H. Lee Moffitt Cancer Center & Research Institute, Tampa, Florida, United States of America; University of South Florida, United States of America

## Abstract

Angiogenesis has been shown to be associated with prostate cancer development. The majority of prostate cancer studies focused on individual single nucleotide polymorphisms (SNPs) while SNP-SNP interactions are suggested having a great impact on unveiling the underlying mechanism of complex disease. Using 1,151 prostate cancer patients in the Cancer Genetic Markers of Susceptibility (CGEMS) dataset, 2,651 SNPs in the angiogenesis genes associated with prostate cancer aggressiveness were evaluated. SNP-SNP interactions were primarily assessed using the two-stage Random Forests plus Multivariate Adaptive Regression Splines (TRM) approach in the CGEMS group, and were then re-evaluated in the Moffitt group with 1,040 patients. For the identified gene pairs, cross-evaluation was applied to evaluate SNP interactions in both study groups. Five SNP-SNP interactions in three gene pairs (*MMP16+ ROBO1*, *MMP16+ CSF1*, and *MMP16+ EGFR*) were identified to be associated with aggressive prostate cancer in both groups. Three pairs of SNPs (rs1477908+ rs1387665, rs1467251+ rs7625555, and rs1824717+ rs7625555) were in *MMP16* and *ROBO1*, one pair (rs2176771+ rs333970) in *MMP16* and *CSF1*, and one pair (rs1401862+ rs6964705) in *MMP16* and *EGFR*. The results suggest that *MMP16* may play an important role in prostate cancer aggressiveness. By integrating our novel findings and available biomedical literature, a hypothetical gene interaction network was proposed. This network demonstrates that our identified SNP-SNP interactions are biologically relevant and shows that EGFR may be the hub for the interactions. The findings provide valuable information to identify genotype combinations at risk of developing aggressive prostate cancer and improve understanding on the genetic etiology of angiogenesis associated with prostate cancer aggressiveness.

## Introduction

Prostate cancer accounts for 29% of cancer incidence and 9% of cancer deaths and it is the most common cancer and the second leading cause of cancer death in American men in 2012 [1]. Prostate cancer has a substantial clinical heterogeneity. Physicians therefore often have difficulty distinguishing between patients who will develop indolent and aggressive tumors at the time of a prostate cancer diagnosis [2]. For prostate cancer patients with a low risk, conservative management and treatment are recommended because an indolent course over a long period of time may be observed. Several features (such as prostate specific antigen, clinical stage and tumor grade) have been used to classify high-risk patients who need immediate therapy and the low risk patients who need conservative treatment. When using the existing features, approximately 20% of these low-risk prostate cancer patients died due to conservative treatment [3]. Thus, there is an urgent need for identifying biomarkers in order to improve prediction accuracy of prostate cancer aggressiveness.

Angiogenesis is a biological process that involves the division and migration of endothelial cells, resulting in microvasculature formation [4], [5]. The formation of blood vessels is important for organ development during embryogenesis and continues to contribute to organ growth after birth. During adulthood, most blood vessels remain quiescent and angiogenesis is limited to the cycling ovary and in the placenta during pregnancy [4], [5], [6]. Nonetheless, endothelial cells maintain their ability to divide rapidly into blood vessels in response to physiological stimuli, such as hypoxia, and angiogenesis is reactivated during wound healing and repair [4], [5], [7]. The process of postnatal angiogenesis is regulated by a continuous interplay (that establishes a balance) of stimulators such as vascular endothelial growth factor (VEGF), basic fibroblast growth factor (bFGF), epidermal growth factor (EGF), interleukins (ILs), transforming growth factor beta (TGF-β), tumor necrosis factor alpha (TNF-α), platelet derived growth factor (PDGF), and matrix metalloproteinases (MMPs) and inhibitors such as endostatin, platelet factor-4, tumastin, thrombospondin-1, plasminogen activator inhibitor-1 and angiostatin [4], [5], [6], [7], [8]. However, in many disorders including prostate cancer, the balance between stimulators and inhibitors is tilted to favor stimulators, resulting in an “angiogenic switch” [9], [10]. The so-called “angiogenic switch” may result from changes in the expression levels of genes in the angiogenesis pathway.

Single nucleotide polymorphisms (SNP) in angiogenesis genes may alter gene expression and influence the process of angiogenesis in prostate cancer and inhibited tumor growth in animal models [11], [12]. Indeed, several SNPs in angiogenesis genes that affect gene expression have been identified. These variants may potentially contribute to inter-individual variation in the risk and progression of prostate tumors [13]. Furthermore, angiogenesis is shown to be associated with the Gleason score, tumor stage, progression, metastasis and survival among prostate cancer patients [14], [15].

Although the number of studies for evaluating the role of SNPs in angiogenesis genes is limited, several of the studies support the association between angiogenesis and prostate cancer aggressiveness. So far, results from several candidate gene and genome-wide association (GWA) studies suggest that SNPs in the angiogenesis pathway may be important in prostate cancer progression and aggressiveness. In the candidate gene studies, *VEGF* -1154A and -634C alleles were associated with an increased risk of higher tumor grade [16]. Jacobs *et al*. (2008) evaluated 58 SNPs in nine angiogenesis genes and found that three correlated SNPs (rs1477017, rs17301608, and rs11639960) in the *MMP2* were associated with overall and advanced prostate cancer [17]. Additionally, men with the *IL-10* 819 TT genotype tended to have a higher risk of developing a high-grade prostate cancer [18]. In a GWA study, Thomas *et al.* observed that a nonsynonymous SNP (rs4072111) that changes a serine to proline in *IL-16* was significantly associated with an increased risk of aggressive cancer [19]. Another GWA study observed significant associations between aggressive prostate cancer and three intergenic SNPs (rs11199874, rs10749408 and rs10788165) that span a 590 kb region on chromosome 10q26 that encompasses *FGFR2*, an angiogenesis gene [20]. Penney observed associations with mortality for SNPs in *IL-18* (rs360729, and rs243908) and *IL-11* (rs12709950) in their stage one scan, but none were replicated in the stage two scan [21].

In order to comprehensively evaluate genetic variations in angiogenesis genes associated with prostate cancer aggressiveness, effects of both individual SNPs and SNP-SNP interactions were examined. The majority of current studies are focused on evaluating individual SNP effects; however, one-to-one associations may not be sufficient to explain the complexity of disease causality. It has recently been established that gene-gene/SNP-SNP interactions may have a higher impact on unveiling causality of complex diseases [22], [23], [24], [25]. Pure SNP-SNP interactions, i.e., those with minor or no significant individual SNP effects, were reported in several diseases, such as breast cancer [26], [27], prostate cancer [28], and rheumatoid arthritis [29].

To overcome the challenges in high-dimensional data, we searched SNP-SNP interactions using the TRM approach, a two-stage Random Forests plus Multivariate Adaptive Regression Splines (MARS). Conventional studies use an additive model for SNPs, and search for pair-wise SNP interactions using logistic regression. This approach is not sufficient because an additive model assumption may not be valid. It has been shown that the genetic model selection has a great impact on the detection power of associations [28]. In some studies, pure SNP interactions are overlooked because only SNPs with a significant or marginal main effect are taken into consideration. For improving the prediction ability of complex disease, identifying appropriate genetic models (such as dominant and recessive) and considering gene-gene interactions in the association studies are suggested [30]. This TRM approach, which takes different inheritance models and interactions into account in both screening and interaction pattern searching steps, has been shown to be powerful in detecting SNP interactions in a large-scale genetic variation study [31].

## Materials and Methods

Two groups were used in this study. The CGEMS group was used as the primary data set to identify SNP-SNP interactions associated with prostate cancer aggressiveness. The significant results identified in the CGEMS data were then re-evaluated using the Moffitt data. All individuals in our analysis were men with European descent because data were available on men with European descent in the CGEMS dataset. Only one common demographic variable, age at enrollment, was available in the two study groups. However, due to the study design difference, the variables of age at enrollment in the two study groups are not comparable. For the prostate cancer patients, the date of enrollment was prior to prostate cancer diagnosis in the CGEMS study (a nested case-control study within a prospective cohort study), but it was after cancer diagnosis in the Moffitt cohort (a case only study). Thus, our analyses were based on unadjusted results.

### CGEMS Population

There were 1,151 prostate cancer patients (659 aggressive and 492 non-aggressive patients) in the CGEMS prostate cancer genome-wide data set. The participants were selected from the Prostate, Lung, Colon and Ovarian (PLCO) Cancer Screening Trial enrollment between 1993 and 2003 [32]. There were 12%, 55% and 33% patients in the age group of <60, 60–69 and > = 70 years-old, respectively, at enrollment of the PLCO cohort study. The whole data contained approximately 550,000 SNPs genotyped with Illumina HumanHap300 and Illumina HumanHap250. Patients with Gleason scores ≥7 or ≥stage III were considered to have aggressive prostate cancer. We identified genes encoding proteins involved in or related to angiogenesis through searching published literature (PubMed) and public pathway database (Cancer Genome Anatomy Project, Kyoto Encyclopedia of Gene and Genomes and Gene Ontology). A total of 2,653 SNPs in the 161 angiogenesis genes were examined. The Hardy-Weinberg equilibrium was examined in the control group (n = 1,101), which were not included in this study. After excluding two SNPs without following the Hardy-Weinberg equilibrium (p-value<10^−4^), a total of 2,651 SNPs were applied for further analyses. Linkage disequilibrium among all testing SNPs was examined based on r^2^ using the Haploview Tagger [33]. After selecting one SNP in each pair with strong linkage disequilibrium of r^2^>0.8, a total of 2,177 SNPs were included for interaction analyses.

### Moffitt Population

The Moffitt group was used in the cross-evaluation of SNPs in angiogenesis genes associated with prostate cancer aggressiveness. The Moffitt population consisted of a historical cohort of 1,040 prostatectomy cases treated at the Moffitt Cancer Center from 1986 to 2003. We identified 437 aggressive cases and 603 non-aggressive cases based upon the same prostate cancer aggressiveness criteria used in the CGEMS group. There were 49%, 42% and 8% patients in the age group of <60, 60–69 and > = 70 years-old, respectively, at enrollment of the Moffitt study. There were 681 angiogenesis SNPs genotyping using the Illumina GoldenGate™ assay (Illumina, San Diego, CA). The study protocol was approved by the Institutional Review Board of the University of South Florida (Tampa, FL).

### Analysis of Individual SNP Effects

In the CGEMS group, three inheritance models (dominant, recessive and additive model) were assessed using logistic regression models, and the best model was selected based on the minimum p-value for each SNP. False discovery rate (FDR) q-value [34] was calculated for adjusting for multiple comparisons. The significant SNPs with a p-value less than 0.05 in the CGEMS were then re-assessed in the Moffitt group. For consistency, the same genetic model with the minimal p-value in the CGEMS group was applied in the Moffitt group for each SNP. The main effects of SNPs involved in the significant interactions were also evaluated.

### Cross-evaluation of SNP-SNP Interactions

For exploring SNP-SNP interactions, the TRM, two-stage Random Forests plus MARS, was applied [31]. In the first stage of the TRM approach, the top candidate SNPs were selected based on minimizing the out-of-bag (OOB) classification error rate using the unscaled permutation accuracy importance index in Random Forests. The candidate SNPs selected in the first stage were explored up to two-way interactions associated with prostate cancer aggressiveness using MARS. In the first stage, we used the default of 5,000 trees to build the first forest and 2,000 trees for all additional forests using the varSelRF R package. The number of randomly selected predictors was set at 47, the square root of the number of predictors. Among all fitted forests, the final set of variables was selected based on the smallest number of variables whose error rate is less than one standard error of the minimum OOB error rate. In MARS, the maximum basis functions of 30 was applied to explore SNP-SNP interaction patterns among the top candidate SNPs. Ten-fold cross-validation was used to select the degree of freedom charged per basis function.

Because Random Forests does not allow for missing values, the sporadically missing genotyped data were imputed using IMPUTE version 2.0 with the HapMap3 CEU+TSI data as the reference population. Among 2,177 SNPs for interaction search, the missing data rate was low: the median missing rate was 2.6% and maximum was 5.6%. In order to preserve all SNPs in the analyses, our interaction analyses were based on the combined dataset composed of the original genotyped data and the imputed data. The individual SNP effects were evaluated using the original data.

The TRM approach does not provide variable significance using p-values, so the bootstrap method was applied for selecting factors in the final model and reducing false positive findings. The bootstrap frequencies of a null model, no association between the simulated outcome and the testing SNPs, were applied for setting the cut-point of variable selection in TRM. In this null model, we independently generated a binary outcome variable with 659 subjects in one group and 492 subjects in the other group, which was consistent with the aggressiveness status in the CGEMS data set. We obtained 500 bootstrap samples by sampling with replacement from the null model. For each identified factor (individual effects or interactions), the false positive frequencies based on 500 bootstrap samples were calculated. The 95% percentile of the bootstrap frequencies in the false positive factors was 4.2%. For conservative purposes, we kept only those with a bootstrap frequency greater than 5%. For easy interpretation, the identified factors were included in the multivariable logistic regression for obtaining odds ratio and their 95% confidence intervals.

**Figure 1:**
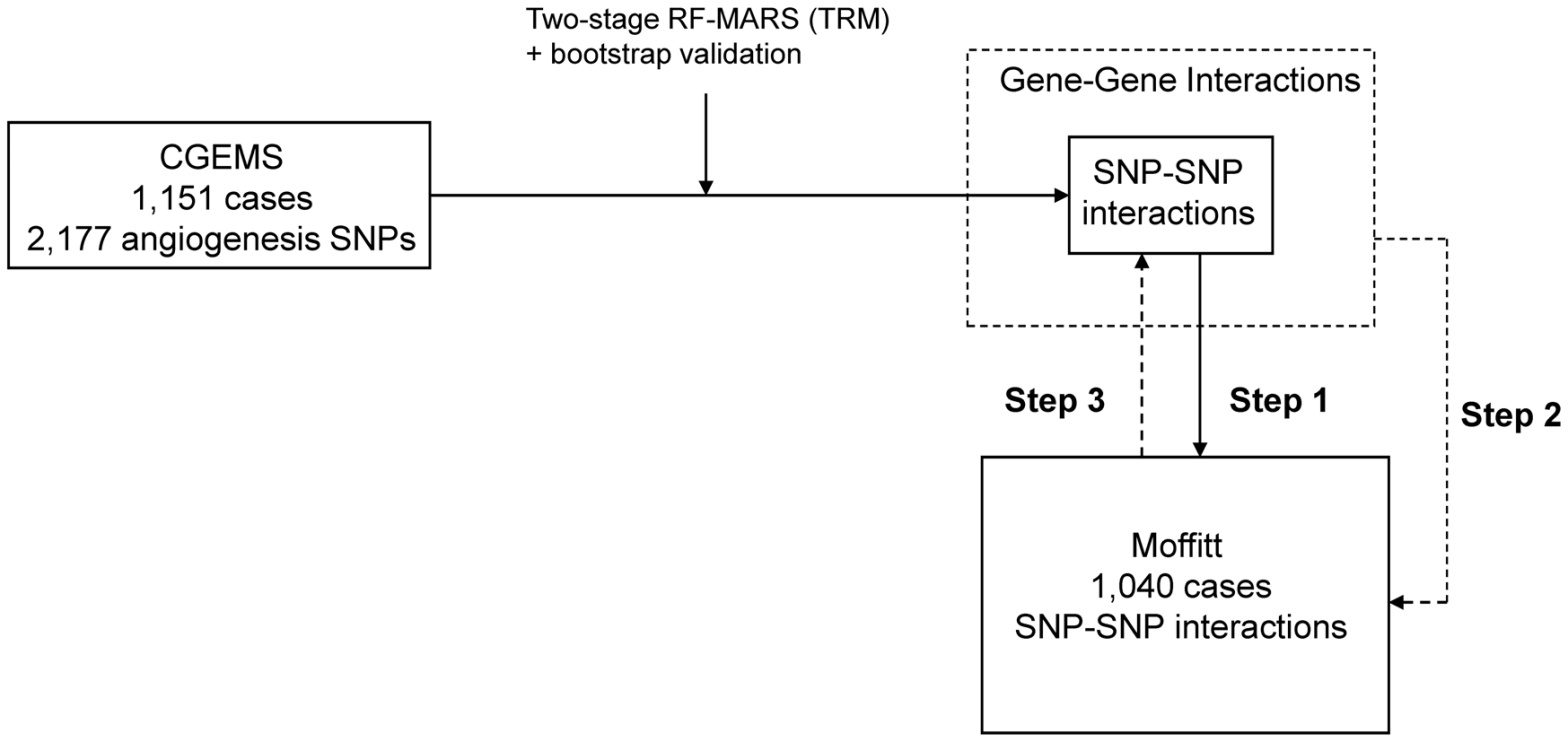
Flow chart of SNP-SNP interaction cross-evaluation. In Step 1, SNP-SNP interactions identified in the CGEMS group were re-assessed in the Moffitt group. In Step 2, all possible two-way SNP-SNP interactions of the identified gene pairs were evaluated in the Moffitt group. In Step 3, the identified SNP interactions from the Step 2 were re-evaluated in the CGEMS group.

The flow chart of the cross-evaluation for detecting SNP-SNP interactions is shown in Figure 1. In Step 1, the SNP-SNP interactions identified in the CGEMS group, treated as a training set, were re-assessed in the Moffitt group. Among the CGEMS identified gene pairs (gene-gene interactions), we were also interested in exploring whether other SNP-SNP interactions could be detected in the independent Moffitt group. In Step 2, we further searched all possible two-way SNP-SNP interactions of the identified gene pairs (such as *MMP16*+ *ROBO1*) in the Moffitt group. In Step 3, the identified SNP interactions from the Step 2 were re-evaluated in the CGEMS group. The best “interaction patterns” (such as dominant-dominant model) were detected separately in both groups using MARS. Additionally, we checked whether the identified interaction models were better than the models with their main effects only by using the stepwise logistic regressions. Data analyses were performed using SAS 9.3. TRM was performed using MARS 2.0 (Salford Systems, San Diego, USA), and R package of randomForest and varSelRF.

## Results

In the Cancer Genetic Markers of Susceptibility (CGEMS) group, we evaluated the main effect of 2,651 angiogenesis SNPs associated with prostate cancer aggressiveness status (yes/no) using logistic regression models. There were 279 SNPs in 75 genes with a raw p-value less than 0.05. Among these SNPs, the largest FDR q-value was 0.053. This indicates that less than 15 false positive findings were expected among the top selected SNPs. These significant SNPs were then assessed in the Moffitt group. Among these 279 SNPs, 160 SNPs were available in the Moffitt data. Four SNPs in three genes (*COL4A3*, *PDGFD* and *ELK3*) were associated with prostate cancer aggressiveness in both the CGEMS and Moffitt groups with a p-value less than 0.05. Two SNPs (rs10498214 and rs6436661) in the *COL4A3* were significantly associated with prostate cancer aggressiveness. Those with the CC genotype compared with CT and TT genotype in rs10498214 tended to have a higher risk of aggressive prostate cancer (odds ratio (OR) = 1.63 and p-value = 0.028 for CGEMS; OR = 1.53 and p-value = 0.047 for Moffitt). The CC and CT genotype of rs6436661 in the *COL4A3* was negatively associated with prostate cancer aggressiveness (OR = 0.74, p-value = 0.040 for CGEMS; OR = 0.71, p-value = 0.034 for Moffitt). Men with the CC genotype in rs488753 (*PDGFD*) were more likely to develop aggressive prostate cancer than those with the CT and TT genotype (OR = 1.47, p-value = 0.035 for CGEMS; OR = 1.45, p-value = 0.031 for Moffitt). The CC and CT genotype of rs2268509 in the *ELK3* was positively associated with prostate cancer aggressiveness (OR = 1.29, p-value = 0.047 for CGEMS; OR = 1.57, p-value = 0.002 for Moffitt).

**Figure 2:**
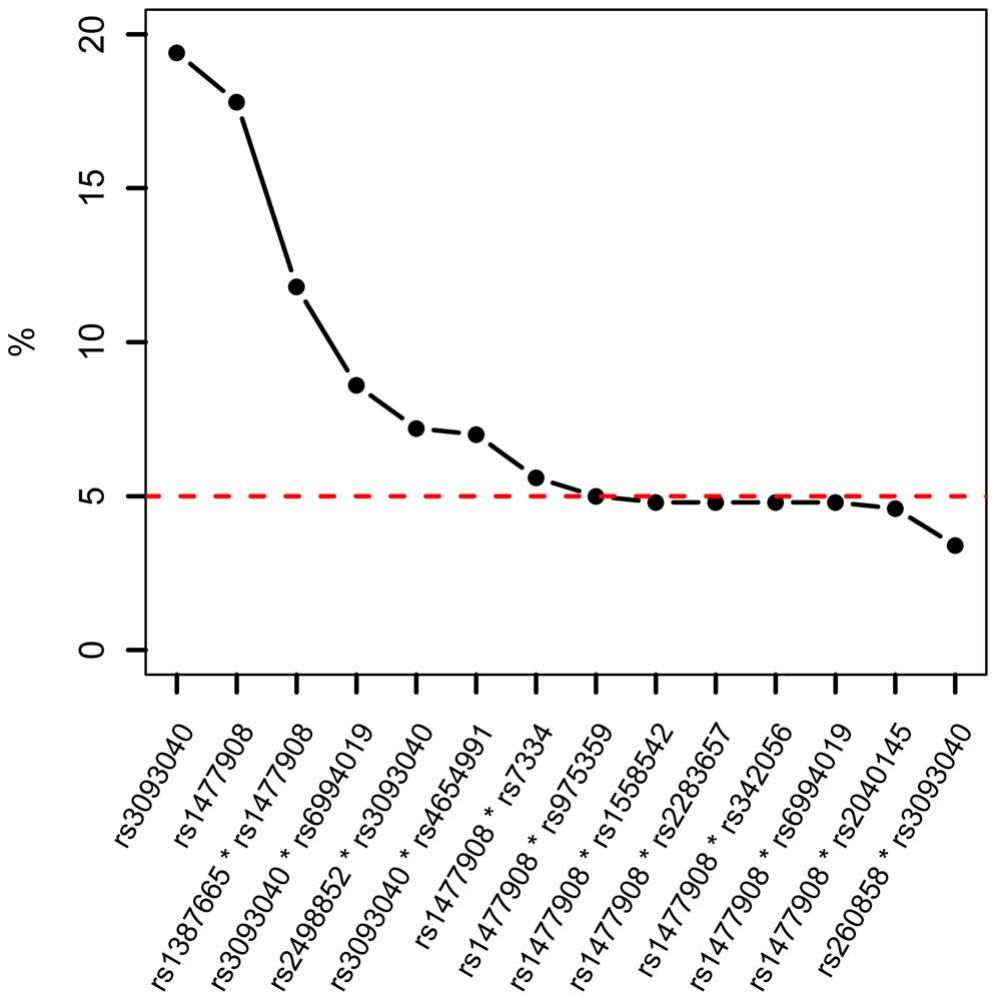
Frequencies based on 500 bootstrap samples of factors selected by the two-stage Random Forests plus MARS (TRM). For reducing false positive findings, the bootstrap method was applied for selecting factors in the final model. We obtained 500 bootstrap samples by sampling with replacement from the null model. For each identified factor, the false positive frequencies based on the 500 bootstrap samples were calculated. The factors with a bootstrap frequency greater than 5% were included in the multivariable model (Table 1).

The SNP interactions in angiogenesis genes were evaluated using the TRM approach in the CGEMS group. A total of 14 factors were selected using the TRM approach (Figure 2). Two main effects of rs3093040 (in *CSF1*) and rs1477908 (in *MMP16*) were selected, and 12 two-way SNP-SNP interactions were identified. For selecting factors in the final model and internal validation, the bootstrap method was applied. Using a bootstrap frequency of 5% as a cut-point, the top seven factors were selected: two main effects and five interacting SNP pairs. The two main effects were the core factors in these SNP pairs. Among the five interacting SNP pairs, rs1477908 was involved in two SNP pairs and rs3093040 was included in the other three SNP pairs. The five gene pairs of the identified SNP-SNP interactions were *MMP16+ ROBO1, MMP16+ CSF1, CSF1+ FBLN5, CSF1+ HSPG2*, and *MMP16+ EGFR,* which in a rank order based on the bootstrap frequencies. As shown in Table 1, we included these important factors in a multivariable logistic model, and all factors were highly significant (p-value range: 0.015–0.0001). These results remained similar after adjusting age (results not shown).

**Table 1:** Model of prostate cancer aggressiveness using the CGEMS group.

Variables	Coefficient (SE) a	p-value
rs1477908 (AG/GG vs. AA)	−0.55 (0.14)	0.0001
rs3093040 (GG/GA vs. AA)	0.58 (0.20)	0.0037
rs1477908+ rs1387665 (AA+AA vs. others)	−0.59 (0.17)	0.0005
rs6994019+ rs3093040 (GG+GG/GA vs. others)	0.47 (0.13)	0.0005
rs3093040+ rs2498852 (GG/GA+GG vs. others)	−0.55 (0.15)	0.0003
rs3093040+ rs4654991 (GG/GA+TC/CC vs. others)	−0.34 (0.14)	0.0145
rs1477908+ rs7334 (AG/GG+ AA vs. others)	−1.76 (0.64)	0.0064

a standard error, based on multivariable logistic model.

Although these five SNP-SNP interactions were significant in the multivariable model, we further evaluated whether these SNP pairs were independent predictors for prostate cancer aggressiveness. The five interacting SNP pairs were fitted individually in a logistic regression model. In each 2-way interaction in Table 2, the reference group was labeled as OR = 1. We defined those with OR>1 as the risk group and those with OR<1 as the protective group. For easy comparison of SNP interaction patterns between the two study groups, the patterns were presented using 3×3 tables in the supplement (Tables S1, S2, S3). These five SNP pairs were significantly associated with prostate cancer aggressiveness in the CGEMS group. In particular, the top two SNP-SNP interactions were rs1477908 (*MMP16*)+rs1387665 (*ROBO1*) and rs6994019 (*MMP16*)+rs3093040 (*CSF1*). Prostate cancer patients with the AA+AG/GG genotype combination of the SNP pair of rs1477908 and rs1387665 were more likely to develop aggressive prostate cancer (OR = 1.83, p-value = 0.0002) than those with the genotype combination of AA+ AA. Patients with the GG/GA genotype in rs3093040 were more likely to have an aggressive prostate cancer than those with the AA genotype, but this effect was significantly modified by rs6994019. Among those with the GG/GA genotype in rs3093040, men with the GG genotype of rs6994019 were more likely to develop aggressive prostate cancer (OR = 2.22, p-value = 1.7*10^−5^) than those with the AA genotype of rs3093040; nevertheless, this positive association was not significant for those with the GT/TT genotype of rs6994019 (OR = 1.36, p-value = 0.096).

**Table 2:** SNP-SNP interactions in angiogenesis genes associated with prostate cancer aggressiveness in the CGEMS and Moffitt group.

Gene-gene	SNP-SNP interaction^a^		CGEMS^b^			Moffitt^b^	
interaction		Genotypecombination	OR (95% CI)	p-value	Genotypecombination	OR (95% CI)	p-value
*MMP16+ ROBO1*	rs1477908(A/G)+rs1387665(A/G)^c1,^ d	AA+AA	1		AA+AA/AG	1	
		AG/GG+ all	0.96 (0.68−1.37)	0.8337	AG/GG+ all	1.07 (0.81−1.43)	0.6263
		AA+AG/GG	1.83 (1.33−2.53)	0.0002	AA+GG	1.39 (0.98−1.97)	0.0651
	rs1467251(G/A)+ rs7625555(G/A)^d^	all+AA	1		AA+GG/GA vs. others	0.29 (0.10−0.85)	0.0239
		GG+GG/GA	0.82 (0.61−1.09)	0.1674			
		GA/AA+ GG/GA	0.59 (0.42−0.82)	0.0017			
	rs1824717(A/G)+ rs7625555(G/A)^d^	AA+ AA vs. others	1.91 (1.18−3.08)	0.0086	AA/AG+ GG	1	
					GG+all	1.43 (0.96−2.13)	0.0762
					AA/AG+ GA/AA	1.59 (1.13−2.24)	0.0080
*MMP16+ CSF1*	rs6994019(G/T)+ rs3093040(G/A)^c2^	All+AA	1		N/A		
		GT/TT+ GG/GA	1.36 (0.95−1.97)	0.0955			
		GG+GG/GA	2.22 (1.54−3.19)	1.7*10^−5^			
	rs2176771(A/C)+ rs333970(A/C)^d^	AA+ AC/CC vs. others	1.52 (1.19−1.94)	0.0007	AC/CC+ CC vs. others	0.50 (0.26−0.95)	0.0339
*CSF1+ FBLN5*	rs3093040(A/G)+ rs2498852(A/G)^c3^	AA+all	1		N/A		
		AG/GG+ GG	1.19 (0.79−1.77)	0.4086			
		AG/GG+ AA/AG	2.01 (1.42−2.85)	8.8*10^−5^			
*CSF1+ HSPG2*	rs3093040(A/G)+ rs4654991(T/C)^c4^	AA+all	1		N/A		
		AG/GG+ TT	1.99 (1.40−2.84)	1.3*10^−4^			
		AG/GG+ TC/CC	1.38 (0.94−2.02)	0.1007			
	rs3093037(G/A)+ rs7556412(A/G)	N/A			All+AG/GG	1	
					AA+AA	2.48 (0.84−7.34)	0.1012
					GG/GA+ AA	0.76 (0.59−0.99)	0.0376
	rs3093037(G/A)+ rs2290501(A/C)	N/A			GG+CC vs. others	0.58 (0.35−0.96)	0.0356
*MMP16+ EGFR*	rs1477908(A/G)+ rs7334(C/A)^c5^	AA+all	1		N/A		
		AG/GG+ AA	0.11 (0.03−0.39)	0.0006			
		AG/GG+ CC/CA	0.67 (0.52−0.87)	0.0027			
	rs1401862(G/A)+ rs6964705(C/A)^d^	GA/AA+ AA vs. others	0.58 (0.38−0.88)	0.0107	GG/GA+ all	1	
					AA+CC	1.55 (0.52−4.66)	0.4316
					AA+CA/AA	0.23 (0.08−0.68)	0.0074
	rs10504853(A/G)+ rs17172446(G/A)	AA+ GA/AA vs. others	1.06 (0.80−1.41)	0.6677	AA+ GA/AA vs. others	1.50 (1.13−2.00)	0.0051
	rs1477908(A/G)+ rs17172446(G/A)	AA+ all vs. others	1.62 (1.26−2.10)	0.0002	AA/AG+ GG vs. others	0.69 (0.54−0.89)	0.0037

a SNP(major/minor allele).

b all: all three genotypes in the SNP; others: genotype combinations of the two SNPs other than the specified genotype.

c1–c5 top 1 to top 5 identified SNP-SNP interactions using the TRM approach in CGEMS.

d similar interaction pattern in the CGEMS and Moffitt group.

These 5 important SNP-SNP interactions were further evaluated using the Moffitt group (Step 1 in Figure 1). Only one SNP-SNP interaction of rs1477908 (*MMP16*) and rs1387665 (*ROBO1*) was available in the Moffitt group. As shown in Table 2, we observed that the high risk group of developing aggressive prostate cancer was those with the AA and GG genotype in the pair of rs1477908 and rs1387665 (OR = 1.39 and p-value = 0.065) in the Moffitt group. The high risk groups selected in both group are similar: AA+ GG and AA+ AG/GG of rs1477908 and rs1387665 in the Moffitt and CGEMS groups, respectively.

Besides the CGEMS identified SNP-SNP interactions, we explored whether other SNP interactions in the identified gene pairs in the Moffitt group were significantly associated with prostate cancer aggressiveness. Two-way SNP-SNP interactions of the five identified gene pairs (*MMP16+ ROBO1, MMP16+ EGFR, MMP16+ CSF1, CSF1+ FBLN5, and CSF1+ HSPG2*) were searched (Step 2, Figure 1). As shown in Table 2, an additional eight SNP-SNP interactions were detected in the Moffitt group. Two interactions were in the gene pair of MMP16+ ROBO1, three were in the MMP16+ EGFR, one was in *MMP16*+ *CSF1* and two were in the CSF1+HSPG2. Among these eight identified SNP-SNP interactions, six were available in the CGEMS; consequently, they were then re-evaluated (Figure 1, Step 3).

Three gene pairs were observed to have at least one SNP-SNP interaction with a similar interaction pattern in the two study groups. The similar interaction pattern was defined as the identified genotype combinations in the two study groups, which are overlapped and with the same direction in terms of a prostate cancer aggressiveness risk. Three (rs1477908+ rs1387665, rs1467251+ rs7625555, and rs1824717+ rs7625555) were in the gene pair of *MMP16* and *ROBO1*. The interaction of rs1401862 and rs6964705 was in the *MMP16* and *EGFR*, and another SNP pair of rs2176771 and rs333970 was in the *MMP16* and *CSF1*. With the exception of the SNP pair of rs1477908 and rs1387665, additional two SNP pairs (rs1467251+ rs7625555 and rs1824717+ rs7625555) in the gene pair of *MMP16* and *ROBO1* were associated with prostate cancer aggressiveness. In the Moffitt group, prostate cancer patients with the AA+GG/GA genotype in the SNP pair of rs1467251 and rs7625555 had a lower chance of developing aggressive prostate cancer than other genotype combinations in the same SNP pair (OR = 0.29, p-value = 0.024). In the CGEMS group, the low risk group was those with the GA/AA+GG/GA genotype in the same SNP pair (OR = 0.59, p-value = 0.002). As for the interaction of rs1824717 and rs7625555, the high risk group of aggressive prostate cancer in the CGEMS group was the combination of AA and AA genotype of this SNP pair (OR = 1.91, p-value = 0.009),and the high risk group in the Moffitt set was the combination of AA/AG and GA/AA genotype (OR = 1.59, p-value = 0.008). With the *MMP16* and *EGFR*, men with the genotype combination of GA/AA and AA in a SNP pair of rs1401862 and rs6964705 tended to be less likely (OR = 0.58, p-value = 0.011) have aggressive prostate cancer than other genotype combinations of the SNP pairs in the CGEMS group. We also observed the significant interaction pattern of this SNP pair in the Moffitt group.

The main effects of SNPs involved in the significant interactions are shown in Table 3. These main effects could not be replicated within the two study groups. Among the 16 SNPs involved in the SNP-SNP interactions associated with prostate cancer aggressiveness in the CGEMS group, 13 SNPs had a p-value less than 0.05 in the univariate analyses. Among the 16 SNPs in the Moffitt group, only rs17172446 had a p-value less than 0.05. We also confirmed that the interaction models listed in Table 2 were better than the main-effect only models (results not shown).

**Table 3:** SNPs involved in significant interactions associated with prostate cancer aggressiveness.

					CGEMS		Moffitt	
SNP	Chromosome	Gene	Major/minor	Model^a^	p-value^b^	OR (95% CI)^c^	p-value^b^	OR (95% CI)^c^
rs3093040	1	*CSF1*	G/A	Rec	**0.0012**	0.57 (0.40−0.80)	–	
rs333970	1	*CSF1*	A/C	Dom	**0.0480**	1.28 (1.00–1.63)	0.5580	1.08 (0.84–1.39)
rs3093037	1	*CSF1*	G/A	Rec	–		0.3449	1.38 (0.71–2.68)
rs7334	7	*EGFR*	C/A	Add	**0.0462**	0.82 (0.68–1.00)	–	
rs6964705	7	*EGFR*	C/A	Dom	0.2813	1.16 (0.89–1.52)	0.2257	0.84 (0.64–1.11)
rs17172446	7	*EGFR*	G/A	Add	0.9106	1.01 (0.83–1.24)	**0.0395**	1.24 (1.01–1.51)
rs2498852	14	*FBLN5*	A/G	Rec	**0.0013**	0.64 (0.49–0.84)	–	
rs4654991	1	*HSPG2*	T/C	Dom	**0.0262**	0.76 (0.59–0.97)	0.8159	0.97 (0.75–1.26)
rs7556412	1	*HSPG2*	A/G	Dom	–		0.0811	1.25 (0.97–1.60)
rs2290501	1	*HSPG2*	A/C	Rec	–		0.1235	0.73 (0.49–1.09)
rs1477908	8	*MMP16*	A/G	Add	**0.0002**	0.65 (0.52–0.81)	0.6761	1.05 (0.83–1.32)
rs6994019	8	*MMP16*	G/T	Dom	**0.0037**	0.71 (0.56–0.89)	0.6054	1.07 (0.83–1.37)
rs1824717	8	*MMP16*	A/G	Add	**0.0038**	0.79 (0.68–0.93)	0.7236	1.03 (0.86–1.24)
rs2176771	8	*MMP16*	A/C	Add	**0.0058**	0.76 (0.62–0.92)	0.1273	0.85 (0.68–1.05)
rs1467251	8	*MMP16*	G/A	Dom	**0.0346**	0.77 (0.60–0.98)	0.7465	1.04 (0.8–1.36)
rs1401862	8	*MMP16*	G/A	Add	**0.0476**	0.81 (0.65–1.00)	0.0773	0.82 (0.66–1.02)
rs10504853	8	*MMP16*	A/G	Rec	0.6939	1.10 (0.68–1.78)	0.3608	0.73 (0.38–1.42)
rs1387665	3	*ROBO1*	A/G	Dom	**0.0040**	1.48 (1.13–1.93)	0.5949	0.93 (0.69–1.23)
rs7625555	3	*ROBO1*	G/A	Rec	**0.0258**	1.36 (1.04–1.79)	0.3401	0.87 (0.65–1.16)

a model with minimum p-value in the CGEMS (Dom: dominant, Rec: recessive, Add: additive model).

b bald: p-value<0.05.

c odds ratio (95% confidence interval).

## Discussion

Our findings identified five SNP-SNP interactions in the angiogenesis genes associated with prostate cancer aggressiveness in the CGEMS group using the novel TRM approach. Five highly significant SNP-SNP interactions (p-value = 2×10^−5^ to 6×10^−4^) with a medium to large effect size were successfully detected even with a relatively small sample size of approximately 1,000. The odds ratios of these SNP interactions were categorized from a medium (OR≥1.5) to large effect size (OR≥2) [35]. The clinical impact of the SNP-SNP interactions may be larger than that for individual SNPs identified in GWA studies. The prediction power of cancer risk for the SNPs identified in GWA studies is limited with the median per-allele OR of 1.22 based on a recent review [30].

**Figure 3:**
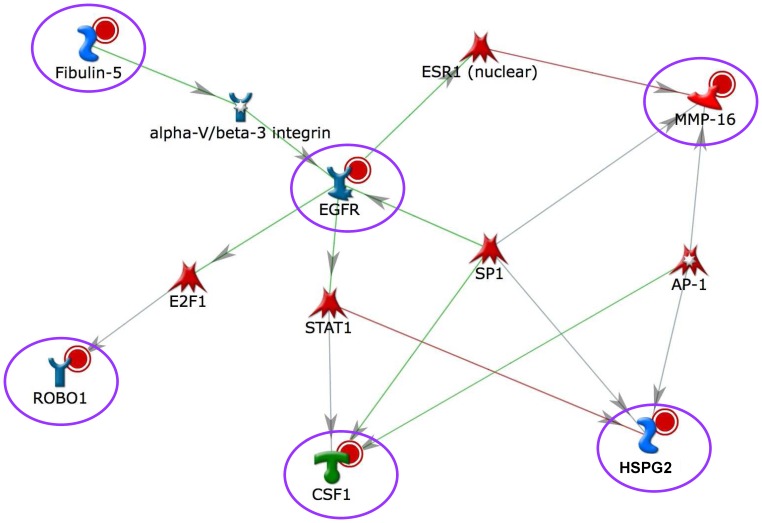
Genetic interaction network based on five interacting gene pairs.^a^. ^a^Five interacting gene pairs: *MMP16+ ROBO1, MMP16+ CSF1, CSF1+ FBLN5, CSF1+ HSPG2*, and *MMP16+ EGFR.* ^b^ Nodes represents the proteins, and lines between nodes indicate interactions between proteins. Green and red lines represent the positive and negative effects, respectively. Proteins of identified genes are denoted by a circle around the nodes.

Our identified gene-gene interactions may be biologically relevant based on the network analysis. The interactions of the five gene pairs (MMP16+ ROBO1, MMP16+ CSF1, MMP16+ EGFR, CSF1+ FBLN5, and CSF1+ HSPG2) were demonstrated using cross-evaluation in the CGEMS and Moffitt groups. Particularly, the former three gene pairs had at least one SNP-SNP interaction with a similar interaction pattern in the two study groups. Among the identified gene pairs, *MMP16* and *CSF1* were involved in several interacting gene pairs, thus a network association was implied. In order to check for biological relevance of the associations and explore the underlying functional mechanism of our identified gene-gene interactions, a hypothetical genetic regulatory network (Figure 3) was proposed. This genetic interaction network was generated based on published protein-protein interactions in *Homo Sapience* using the MetaCore database from GeneGo Inc. The interconnectedness of biochemical process networks of the identified genes showed that the six proteins were involved directly or indirectly in the *EGFR* signaling pathway. It suggested that these genes might be co-regulated by several transcription factors together, such as E2F1, STAT1, ESR1, SP1, and AP-1, and a receptor (integrin). The most prominent protein in the network was EGFR, which interacted with the remaining five proteins that were involved in angiogenesis.

The epidermal growth factor receptor (EGFR) is a critical protein in proliferation of epithelial cells and is involved in oncogenesis. The EGFR binds the epidermal growth factor (EGF) and has been shown to play an important role in regulating prostate cellular growth and function [36], [37], [38]. Our results were also supported by a recent integrative microarray study. Wang *et al.* performed a meta-analysis of 10 prostate cancer microarray expression datasets to identify the common signatures at both the gene and pathway levels associated with prostate cancer risk, and the EGFR pathway was found in nine datasets [39].

The interaction between *MMP16* and *ROBO1*, our top one selection, is promising. *ROBO1* is cleaved by MMPs and translocates into the nucleus of cancer cells, which suggests that ROBO1 may act beyond a receptor as a signaling molecule [40]. Though no specific MMP has been identified yet in the cleavage of ROBO1, our finding may suggest a potential role for MMP16 in the cleavage of ROBO1. These two proteins have also been implicated in prostate cancer and other cancers. MMPs are a multifarious family of proteolytic enzymes involved in tumor growth, invasion and metastasis through the breakdown of extracellular matrix and release of pro-angiogenic factors [41]. The family of mammalian MMPs includes 24 members, but unlike MMP-1, -2 and -9, the role of MMP16 in prostate cancer has not been well investigated. Jung *et al.* reported a down-regulation of *MMP16* in malignant prostate tissues [42]. *MMP16* has been shown to be associated with pancreatic cancer cell migration and invasion [43] and lung development [44]. ROBO1 belongs to a large, single-pass transmembrane cell surface receptors involved in multiple cell processes, including cell migration, myogenesis, leukocyte chemotaxis and tumor angiogenesis [45]. *ROBO1* expression was significantly increased in prostate tumors and hepatocellular carcinoma as compared to normal tissue [46] [47]. It is also frequently methylated and associated with shorter survival in mantle cell lymphoma [48].

Other proteins in the genetic interaction network were reported to be associated with prostate cancer. Colony stimulating factor-1 (CSF1) is a protein that increase tumor angiogenesis [49] and promotes metastatic potential in beast cancer [50]. Although there is no report on a role of CSF1 in prostate cancer, previous studies reported overexpression of serum CSF1 in tumors of several cancer sites, including breast, ovary, and endometrial tissues [49], [51]. Furthermore, the expression of CSF1 in ovarian cancer was shown to be associated with poor outcome [52] Recently, Pyonteck *et al.* observed a potential role in pancreatic cancer. The mouse model of pancreatic cancer without Csf1 gene had a significant decrease in angiogenesis and reduction in tumor number [53]. Previous studies reported that fibulin-5 (FBLN5) suppresses tumorigenesis by inhibiting cell proliferation and angiogenesis by antagonizing VEGF signaling pathway [54], [55], [56], [57]. Wlazlinski *et al.* compared expression of FBLN5 between prostate tumors, benign prostatic tissues and different prostate cancer cell lines. All analyses, microarray, immunohistochemistry (IHC), and RT-PCR consistently suggested down-regulation of *FBLN5* in prostate tumors [58]. Further, FBLN5 was predominantly located in the stroma with a gradient from the periurethral to the peripheral zone, and silenced in tumor tissues [58]. *FBLN5* is known as a target gene for TGF-β [59] and enhance epithelial–mesenchymal transition (EMT) via a MMP-dependent mechanism [60]. Perlecan gene heparan sulfate proteoglycan 2 (HSPG2) regulates the angiogenesis in animal model. *HSPG2* deficient mice showed impaired angiogenesis, delayed wound healing and tumor growth [61]. Later this protein was suggested as one of cancer biomarkers in a few studies [62], [63], [64]. Datta *et al.* demonstrate a positive correlation between expression of HSPG2 and high Gleason tumors [64]. HSPG2 is also required for metastasis and leads to efficient tumor growth and enhancing angiogenesis [65].

Our study demonstrated that frequent inconsistent results of individual SNPs may be partially due to SNP-SNP interactions. Similar SNP-SNP interaction patterns were observed in the majority of our results, but the individual SNP effects for the SNPs involved in the interactions could not be replicated in the two study groups. For the genetic association validation studies, it is well known the individual SNP results are difficult to reproduce. Hirschhorn *et al.* evaluated more than 600 reported associations and found less than 4% of the results were replicable among 166 associations that had been studied more than three times [66]. Furthermore, the gene set identified in the main SNP effect and interaction approaches were totally different in our study. The four SNPs in the three genes (*COL4A3, PDGFD* and *ELK3*) with significant main effects in our two study groups did not overlap with the SNPs with significant interactions. Thus, it is highly recommended to consider both main effects and interactions for comprehensively evaluating gene variations in genetic association studies.

Our study findings (Table 1), generated from the TRM approach by considering multiple SNPs simultaneously, may provide more useful information in building a multivariable prediction model than the pair-wise search approaches, which consider two SNPs at a time. However, it should be noted that our study may not find all SNP-SNP interactions due to a limited sample size of each testing data set and characteristics of the TRM method. Although Random Forests have been shown to perform reasonably well in detecting pure SNP-SNP interactions [67], it still favors SNPs with strong main effects. Larger studies and a combination of multiple analytical approaches are warranted to further test SNP-SNP interactions in angiogenesis genes associated with prostate cancer aggressiveness.

In summary, this study successfully detected the genotype combinations at risk of aggressive prostate cancer and explored the underlying complicated biological associations among angiogenesis genes associated aggressiveness of prostate cancer. The gene network associations based on SNP interactions were also observed in several studies for various diseases [25], [68], [69]. The network constructed based upon our SNP-SNP interaction results indicates novel relationships among critical genes involved in the angiogenesis pathway. More importantly, as Figure 3 illustrates, the interactions between two genes identified in our study can be interpreted as the result of two genes that are co-regulated by a common transcription factor. These findings can be beneficial for providing valuable information to guide follow-up functional analysis as well as potential targets for drug discovery.

## Supporting Information

Table S1 SNP-SNP interactions of *MMP16+ROBO1* and *CSF1+FBLN5* associated with prostate cancer aggressiveness.
(DOC)

Table S2 SNP-SNP interactions of *MMP16+CSF1* and*CSF1+HSPG2* associated with prostate cancer aggressiveness.
(DOC)

Table S3 SNP-SNP interactions of *MMP16+EGFR* associated with prostate cancer aggressiveness.
(DOC)
