# Supplementary material for: SNP-SNP Interaction Network in Angiogenesis Genes Associated with Prostate Cancer Aggressiveness
Source: PLoS One. 2013 Apr 3;8(4):e59688. doi: 10.1371/journal.pone.0059688 (PMC3618555; doi:10.1371/journal.pone.0059688)
Supplement: Table S1 — SNP-SNP interactions of MMP16+ROBO1 and CSF1+FBLN5 associated with prostate cancer aggressiveness. (DOC) [file pone.0059688.s001.doc]

Table S1. SNP-SNP interactions of *MMP16+ROBO1* and *CSF1+FBLN5* associated with prostate cancer aggressiveness

| Training set |  |  | **CGEMS**^a^ | | |  |  |  |  | **Moffitt**^a^ |  |
| --- | --- | --- | --- | --- | --- | --- | --- | --- | --- | --- | --- |
|  | ***MMP16+ ROBO1*** | |  | | |  |  |  |  |  |  |
|  |  |  | rs1387665 (A/G) | | |  |  |  |  | rs1387665 (A/G) |  |
| CGEMS ^C^ | rs1477908 (A/G) ^b^ | AA | AG | | | GG |  | rs1477908 (A/G) ^b^ | AA | AG | GG |
|  | AA | **OR=1** | **1.83 (1.33-2.53)***** | | |  |  | AA | **OR=1** |  | **1.39 (0.98-1.97)^#^** |
|  | AG |  |  | | |  |  | AG |  |  |  |
|  | GG |  | **0.96 (0.68-1.37)** | | |  |  | GG |  | **1.07 (0.81-1.43)** |  |
|  |  |  |  | | |  |  |  |  |  |  |
|  |  |  | rs7625555 (G/A) | | |  |  |  |  | rs7625555 (G/A) |  |
| Moffitt ^C^ | rs1467251 (G/A) | GG | GA | | | AA |  | rs1467251 (G/A) | GG | GA | AA |
|  | GG | **0.82 (0.61-1.09)** |  | | |  |  | GG |  | **OR=1** |  |
|  | GA |  |  | | | **OR=1** |  | GA |  |  |  |
|  | AA | **0.59 (0.42-0.82)**** |  | | |  |  | AA | **0.29 (0.10-0.85)*** |  |  |
|  |  |  |  | | |  |  |  |  |  |  |
|  |  |  | rs7625555 (G/A) | | |  |  |  |  | rs7625555 (G/A) |  |
| Moffitt ^C^ | rs1824717 (A/G) | GG | GA | | | AA |  | rs1824717 (A/G) | GG | GA | AA |
|  | AA |  |  | | | **1.91 (1.18-3.08)**** |  | AA | **OR=1** | **1.59 (1.13-2.24)**** |  |
|  | AG |  | **OR=1** | | |  |  | AG |  |  |  |
|  | GG |  |  | | |  |  | GG |  | **1.43 (0.96-2.13) ^#^** |  |
|  |  |  |  | | |  |  |  |  |  |  |
|  | ***CSF1+ FBLN5*** |  | |  |  | |  |  |  |  |  |
|  |  |  | | rs2498852 (A/G) |  | |  |  |  |  |  |
| CGEMS | rs3093040 (A/G) | AA | | AG | GG | |  |  |  |  |  |
|  | AA |  | | **OR=1** |  | |  |  |  | N/A |  |
|  | AG | **2.01 (1.42-2.85)****** | |  | **1.19 (0.79-1.77)** | |  |  |  |  |  |
|  | GG |  | |  |  | |  |  |  |  |  |

^a^ SNP(major/minor allele); white: reference (OR=1); **gray**: no significant (p>=0.05, #:0.05<p<0.1); **black**: risk (OR>1)/protective (OR<1) group compared with reference, * p<0.05, ** p<0.01, *** p<0.001, **** p<0.0001

^b^ Interaction pattern, using rs1477908 + rs1387665 as an example:

CGEMS: AA+ AA (reference, OR=1); AG/GG+ all (OR=0.96); AA+ AG/GG (OR=1.83)

Moffitt: AA+ AA/AG (reference, OR=1); AG/GG + all (OR=1.07); AA+ GG (OR=1.39)

^C^ similar interaction pattern in the CGEMS and Moffitt group
