# Supplementary material for: SNP-SNP Interaction Network in Angiogenesis Genes Associated with Prostate Cancer Aggressiveness
Source: PLoS One. 2013 Apr 3;8(4):e59688. doi: 10.1371/journal.pone.0059688 (PMC3618555; doi:10.1371/journal.pone.0059688)
Supplement: Table S2 — SNP-SNP interactions of MMP16+CSF1 andCSF1+HSPG2 associated with prostate cancer aggressiveness. (DOC) [file pone.0059688.s002.doc]

Table S2. SNP-SNP interactions of *MMP16+CSF1* and *CSF1+HSPG2* associated with prostate cancer aggressiveness

| Training set |  |  | **CGEMS** ^a^ | | |  |  |  |  | **Moffitt** ^a^ |  |
| --- | --- | --- | --- | --- | --- | --- | --- | --- | --- | --- | --- |
|  | ***MMP16+ CSF1*** | | | | | |  |  | | |  |
|  |  |  | | rs3093040 (G/A) |  | |  |  |  |  |  |
| CGEMS | rs6994019 (G/T) | GG | | GA | AA | |  |  |  |  |  |
|  | GG | **2.22 (1.54-3.19)****** | |  | **OR=1** | |  |  |  | N/A |  |
|  | GT |  | | **1.36 (0.95-1.97) ^#^** |  | |  |  |  |  |  |
|  | TT |  | |  |  | |  |  |  |  |  |
|  |  | | | | | |  |  |  |  |  |
|  |  |  | | rs333970 (A/C) |  | |  |  |  | rs333970 (A/C) |  |
| Moffitt ^C^ | rs2176771 (A/C) ^b^ | AA | | AC | CC | |  | rs2176771 (A/C) ^b^ | AA | AC | CC |
|  | AA |  | | **1.52 (1.19-1.94)***** |  | |  | AA |  |  |  |
|  | AC | **OR=1** | |  |  | |  | AC |  | **OR=1** | **0.50 (0.26-0.95)*** |
|  | CC |  | |  |  | |  | CC |  |  |  |
|  |  |  | |  |  | |  |  |  |  |  |
|  | ***CSF1+ HSPG2*** |  | |  |  | |  |  |  |  |  |
|  |  |  | | rs4654991 (T/C) |  | |  |  |  |  |  |
| CGEMS | rs3093040 (A/G) | TT | | TC | CC | |  |  |  |  |  |
|  | AA |  | | **OR=1** |  | |  |  |  |  |  |
|  | AG | **1.99 (1.40-2.84)***** | | **1.38 (0.94-2.02)** |  | |  |  |  | N/A |  |
|  | GG |  | |  |  | |  |  |  |  |  |
|  |  |  | |  |  | |  |  |  |  |  |
|  |  |  | |  |  | |  |  |  | rs7556412 (A/G) |  |
| Moffitt |  |  | |  |  | |  | rs3093037 (G/A) | AA | AG | GG |
|  |  |  | | N/A |  | |  | GG | **0.76 (0.59-0.99)*** | **OR=1** |  |
|  |  |  | |  |  | |  | GA |  |  |  |
|  |  |  | |  |  | |  | AA | **2.48 (0.84-7.34)** |  |  |
|  |  |  | |  |  | |  |  |  |  |  |
|  |  |  | |  |  | |  |  |  | rs2290501(A/C) |  |
| Moffitt |  |  | |  |  | |  | rs3093037 (G/A) | AA | AC | CC |
|  |  |  | |  |  | |  | GG |  |  | **0.58 (0.35-0.96)*** |
|  |  |  | | N/A |  | |  | GA |  | **OR=1** |  |
|  |  |  | |  |  | |  | AA |  |  |  |

^a^ SNP(major/minor allele); white: reference (OR=1); **gray**: no significant (p>=0.05, #:0.05<p<0.1); **black**: risk (OR>1)/protective (OR<1) group compared with reference, * p<0.05, ** p<0.01, *** p<0.001, **** p<0.0001

^b^ Interaction pattern, using rs2176771 + rs333970 as an example:

CGEMS: AA+ AC/CC vs. reference (OR=1.52)

Moffitt: AC/CC+ CC vs. reference (OR=0.50)

^c^ similar interaction pattern in the CGEMS and Moffitt group
