# Supplementary material for: SNP-SNP Interaction Network in Angiogenesis Genes Associated with Prostate Cancer Aggressiveness
Source: PLoS One. 2013 Apr 3;8(4):e59688. doi: 10.1371/journal.pone.0059688 (PMC3618555; doi:10.1371/journal.pone.0059688)
Supplement: Table S3 — SNP-SNP interactions of MMP16+EGFR associated with prostate cancer aggressiveness. (DOC) [file pone.0059688.s003.doc]

Table S3. SNP-SNP interactions of *MMP16+EGFR* associated with prostate cancer aggressiveness

| Training set |  |  | **CGEMS**^a^ |  |  |  |  | **Moffitt** ^a^ |  |
| --- | --- | --- | --- | --- | --- | --- | --- | --- | --- |
|  | ***MMP16+ EGFR*** | | | |  |  | | |  |
|  |  |  | rs7334 (C/A) |  |  |  |  |  |  |
| CGEMS | rs1477908 (A/G) | CC | CA | AA |  |  |  |  |  |
|  | AA |  | **OR=1** |  |  |  |  | N/A |  |
|  | AG | **0.67 (0.52-0.87)**** |  | **0.11 (0.03-0.39)***** |  |  |  |  |  |
|  | GG |  |  |  |  |  |  |  |  |
|  |  | | | |  |  | | |  |
|  |  |  | rs6964705 (C/A) |  |  |  |  | rs6964705 (C/A) |  |
| Moffitt ^C^ | rs1401862 (G/A) ^b^ | CC | CA | AA |  | rs1401862 (G/A) ^b^ | CC | CA | AA |
|  | GG |  |  |  |  | GG |  | **OR=1** |  |
|  | GA |  | **OR=1** | **0.58 (0.38-0.88)*** |  | GA |  |  |  |
|  | AA |  |  |  |  | AA | **1.55 (0.52-4.66)** | **0.23 (0.08-0.68)**** |  |
|  |  |  |  |  |  |  |  |  |  |
|  |  |  | rs17172446 (G/A) |  |  |  |  | rs17172446 (G/A) |  |
| Moffitt | rs10504853 (A/G) | GG | GA | AA |  | rs10504853 (A/G) | GG | GA | AA |
|  | AA |  | **1.06 (0.80-1.41)** |  |  | AA |  | **1.50 (1.13-2.00)**** |  |
|  | AG | **OR=1** |  |  |  | AG | **OR=1** |  |  |
|  | GG |  |  |  |  | GG |  |  |  |
|  |  | | | |  |  | | |  |
|  |  |  | rs17172446 (G/A) |  |  |  |  | rs17172446 (G/A) |  |
| Moffitt | rs1477908 (A/G) | GG | GA | AA |  | rs1477908 (A/G) | GG | GA | AA |
|  | AA |  | **1.62 (1.26-2.10)***** |  |  | AA | **0.69 (0.54-0.89)**** |  |  |
|  | AG |  | **OR=1** |  |  | AG |  | **OR=1** |  |
|  | GG |  |  |  |  | GG |  |  |  |

^a^ SNP(major/minor allele); white: reference (OR=1); **gray**: no significant (p>=0.05, #:0.05<p<0.1); **black**: risk (OR>1)/protective (OR<1) group compared with reference, * p<0.05, ** p<0.01, *** p<0.001, **** p<0.0001

^b^ Interaction pattern, using rs1401862 + rs6964705 as an example:

CGEMS: GA/AA +AA vs. reference (OR=0.58)

Moffitt: GG/AG + all (reference); AA+CC (OR=1.55); AA+ CA/AA (OR=0.23)

^c^ similar interaction pattern in the CGEMS and Moffitt group
